# The effect of pelvic movements of a gait training system for stroke patients: a single blind, randomized, parallel study

**DOI:** 10.1186/s12984-021-00964-7

**Published:** 2021-12-28

**Authors:** Choonghyun Son, Anna Lee, Junkyung Lee, DaeEun Kim, Seung-Jong Kim, Min Ho Chun, Junho Choi

**Affiliations:** 1 The Center for Bionics, Korea Institute of Science and Technology, 5, Hwarang-ro 14-gil, Seungbuk-gu, Seoul, 02792 Republic of Korea; 2 Department of Rehabilitation Medicine, Asan Medical Center, University of Ulsan College of Medicine, 88, Olympic-ro 43-gil, Songpa-gu, Seoul, 05505 Republic of Korea; 3 Department of Electrical and Electonic Engineering, Yonsei university, 50, Yonsei-ro, Seodeamun-gu, Seoul, 03722 Republic of Korea; 4 Hallym University Dongtan Sacred Heart Hospital, 7, Keunjaebong-gil, Hwaseong-si, Gyeonggi-do 18450 Republic of Korea; 5 Department for Biomedical Engineering, Korea University College of Medicine, 73, Goryeodae-ro, Seongbuk-gu, Seoul, 02841 Republic of Korea

**Keywords:** Rehabilitation, Stroke, Exoskeleton, Gait training, Pelvic movement

## Abstract

**Background:**

Aging societies lead to higher demand for gait rehabilitation as age-related neurological disorders such as stroke and spinal cord injury increase. Since conventional methods for gait rehabilitation are physically and economically burdensome, robotic gait training systems have been studied and commercialized, many of which provided movements confined in the sagittal plane. For better outcomes of gait rehabilitation with more natural gait patterns, however, it is desirable to provide pelvic movements in the transverse plane. In this study, a robotic gait training system capable of pelvic motions in the transverse plane was used to evaluate the effect of the pelvic motions on stroke patients.

**Method:**

Healbot T, which is a robotic gait training system and capable of providing pelvic movements in the transverse plane as well as flexion/extension of the hip and knee joints and adduction/abduction of the hip joints, is introduced and used to evaluate the effect of the pelvic movement on gait training of stroke patients. Gait trainings in Healbot T with and without pelvic movements are carried out with stroke patients having hemiparesis.

**Experiment:**

Twenty-four stroke patients with hemiparesis were randomly assigned into two groups and 23 of them successfully completed the experiment except one subject who had dropped out due to personal reasons. Pelvis-on group was provided with pelvic motions whereas no pelvic movement was allowed for pelvis-off group during 10 sessions of gait trainings in Healbot T. Electromyography (EMG) signals and interaction forces as well as the joint angles of the robot were measured. Gait parameters such as stride length, cadence, and walking speed were measured while walking on the ground without assistance of Healbot T after gait training on 1st, 5th, and 10th day.

**Result:**

Stride length significantly increased in both groups. Furthermore, cadence and walking speed of the pelvis-on group were increased by 10.6% and 11.8%. Although interaction forces of both groups except the thighs showed no differences, EMG signals from gluteus medius of the pelvis-on group increased by 88.6% during stance phase. In addition, EMG signals of biceps femoris, gastrocnemius medial, and gastrocnemius lateral of the pelvis-on group increased whereas EMG signals of the pelvis-off group except gastrocnemius lateral showed no difference after gait trainings.

**Conclusion:**

Gait training using a robotic gait training system with pelvic movements was conducted to investigate the effects of lateral and rotational pelvic movements in gait training of stroke patients. The pelvic movements affected to increase voluntary muscle activation during the stance phase as well as cadence and walking speed.

***Clinical trial registration*:**

KCT0003762, 2018-1254, Registered 28 October 2018, https://cris.nih.go.kr/cris/search/search_result_st01_kren.jsp?seq=14310&ltype=&rtype=

## Introduction

As society is getting aged, the number of stroke patients is increasing [1]. By 2030, 70 million stroke patients and 12 million deaths by stroke in the world are anticipated [2, 3]. As a consequence, increased number of stroke patients have suffered from loss of gait function, which is essential for activities of daily living (ADL’s) [4].

Conventional therapies to recover the gait function impose physical load to the therapists as well as economical burden to the patients. In addition, the outcomes of gait training are often limited by inaccurate gait patterns and incorrect assessment of the patients [5, 6]. In order to reduce the burdens, robotic gait training systems have been studied and used [7, 8]. These robotic gait training systems effectively increased the time and intensity of training, which is crucial for better rehabilitation outcomes. Despite the increased time and intensity of training, however, reduced muscle activation was observed if the subjects remained completely passive in those gait training systems [9]. In order to overcome this drawback, several research activities have improved not only by control but also through modification of existing devices or creation of new devices to increase the level of participation of the patients by giving the patients a certain amount of freedom to change trajectories of the gait training systems [10–14].

Further improvements have been made to include pelvic movements in the transverse plane, which were excluded in the early designs due to higher complexity and costs to build the gait training systems. However, since lateral and rotational pelvic movements, which are two of the six determinants of walking [15], are responsible for balancing and weight-shifting during walking and increasing stride length [16–18], it is natural to include those movements for better gait training. Therefore, the lateral and rotational pelvic movements have been added in recently developed robotic gait training systems. Banala et al. added passive joints to a gait training system to allow lateral and rotational pelvic movements [19]. The passive joints including the ones allowing lateral and rotational movements in the transverse plane were held with springs to allow pelvic movements during gait training. In other cases, series elastic actuators were used to actuate an exoskeleton capable of providing translational and lateral movements in the transverse plane as well as joint rotation in the sagittal plane [20–22]. The experiments involving healthy subjects in these systems showed similar muscle activation compared to treadmill walking. However, the effects of the pelvic movements were not studied in depth. FreeD, which was an optional module to allow pelvic movements, was added to the Lokomat to accommodate more physiological gait patterns [18, 23, 24]. When compared to the case without the module, less compensatory movements of the trunk were observed when the pelvic motion was added. Hence, more natural gait patterns were obtained during training with less compensatory movement of the trunk.

In this study, Healbot T, which is a gait training system capable of providing pelvic movements, is introduced. The concept and feasibility of Healbot T was introduced in the previous studies and the effect of the pelvic movements on healthy people using a gait training system called COWALK-I was presented in [25, 26]. Healbot T, which was developed and improved from COWALK-I, was used to study the effect of pelvic movements during gait training of stroke patients with hemiparesis. The patients were randomly divided into two groups and asked to walk in Healbot T. During gait trainings, one group was provided with pelvic movements whereas the other group walked without pelvic movements. The group with pelvic motions showed increased muscle activation during the stance phase while interaction forces at the pelvis showed no significant difference. Stride length in both groups increased whereas cadence and walking speed of the pelvis-on group increased by 10.6% and 11.8%.

## Healbot T

**Fig. 1:**
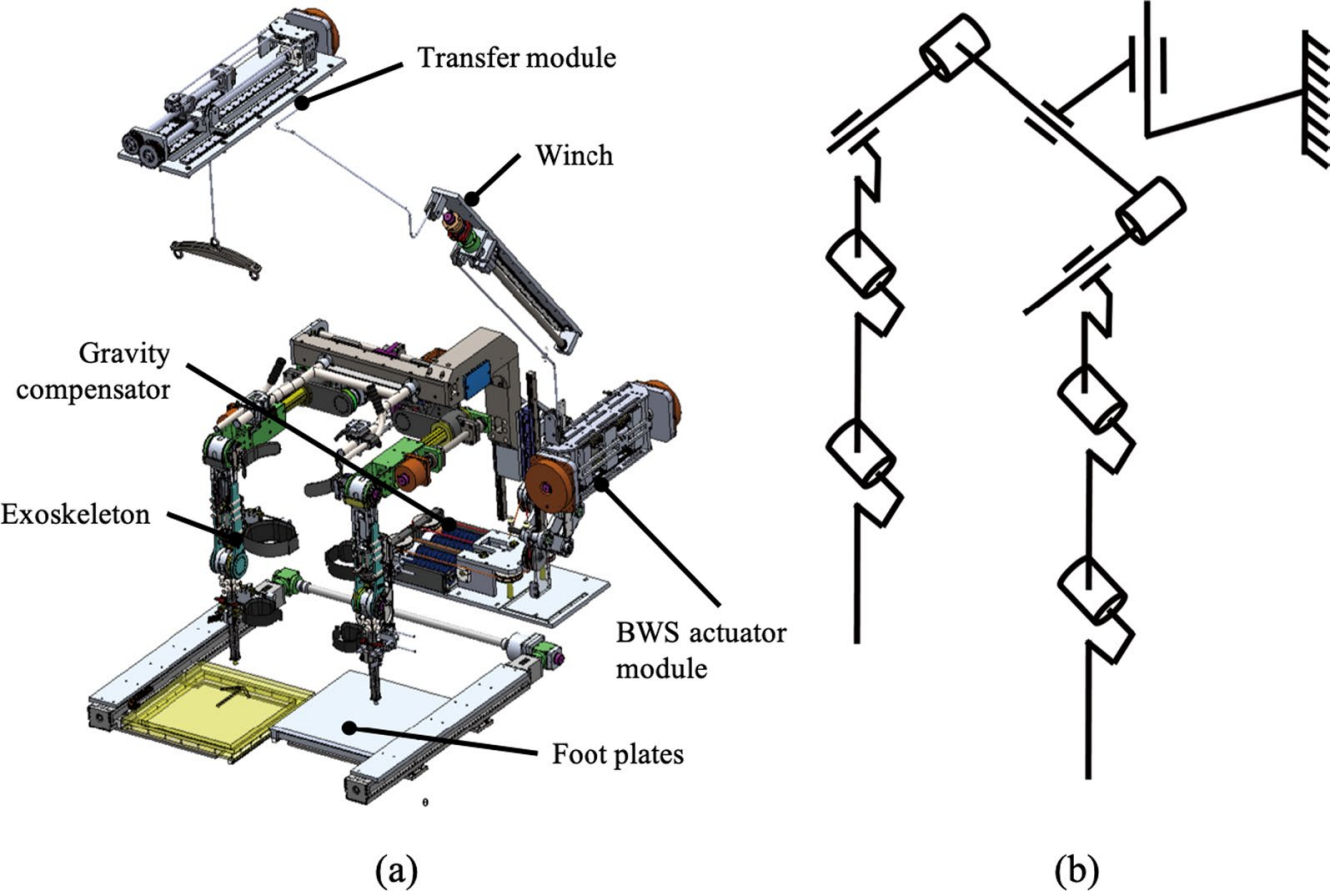
**a** Designed Healbot T. Healbot T consists of a lower limb exoskeleton robot with 9 active DoF and 1 passive DoF, a BWS system, a gravity compensator, and foot plates. **b** Schematics of Healbot T, which has 9 active joints and 1 passive joint for vertical displacement

Healbot T is a gait training system designed for patients with stroke. It consists of an exoskeleton robot, a body weight support (BWS) system, a gravity compensator, and footplates, see Fig. 1.

The exoskeleton robot is designed to provide pelvic movements. It has 9 active joints, which are three prismatic joints for translational and rotational movements in the transverse plane and six rotational joints for adduction/abduction at the hip joint and extension/flexion of the hip and knee joints, and 1 passive joint for vertical displacement. For the rotational joints, six identical BLDC motors (K064050-EY2, Parker) with harmonic gear reducers (SHG-20-100-2SO, HDS) are used. Three prismatic joints are actuated with three identical BLDC motors (K089200-7Y2, Parker) with lead-screws to generate lateral, forward, and rotational pelvic movements in the transverse plane. Positions of the actuated joints are measured using absolute encoders (EQI-1131, Heidenhain) attached to the shaft of the motors.

**Fig. 2:**
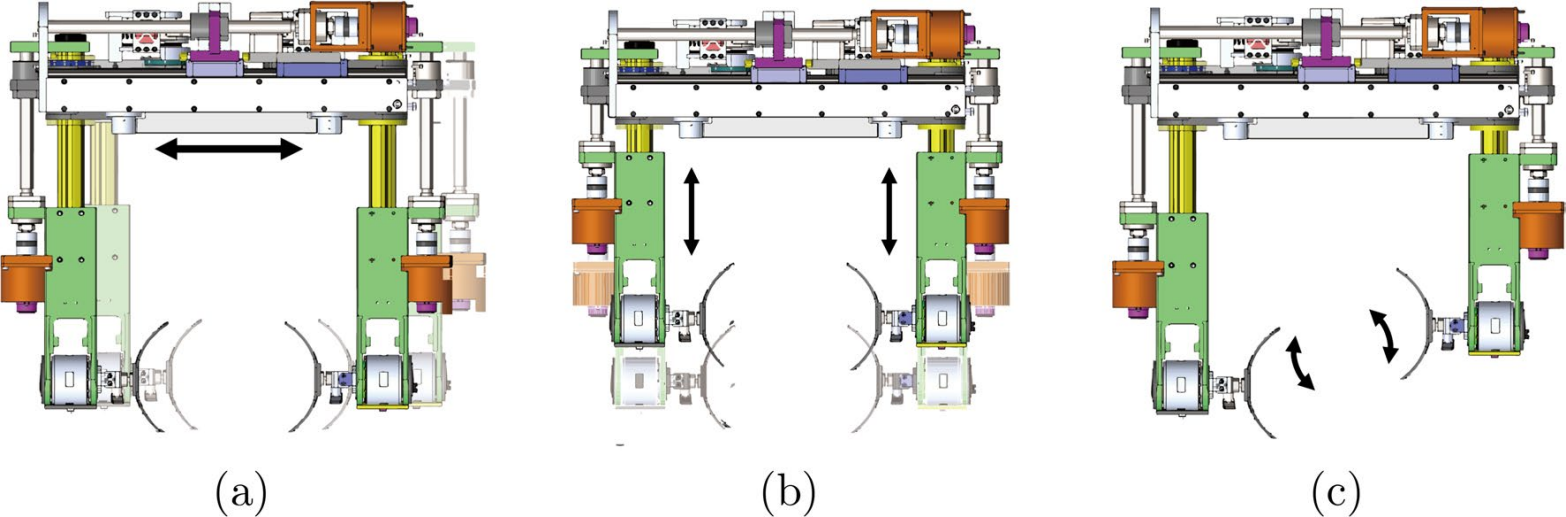
Pelvic movement generated with three prismatic joints **a** Lateral movement, **b** forward movement, **c** rotational movement

Pelvic movements in the transverse plane are generated using three prismatic joints. Lateral movement is generated by the prismatic joint in the center of the exoskeleton. Forward and rotational movements are produced by the prismatic joints at both sides. See Fig. 2.

The braces for securing the patients are located at the pelvis, thighs and calves of the exoskeleton. Force sensors (UMM31-K100, Dacell) are installed between the exoskeleton and the braces to measure the interaction forces. The footplates are synchronized to the horizontal position of the patients. In order to ensure safety, each joint angle is kept within a preset range of motion by mechanical and programmable limits.

The BWS system is designed for supporting the subjects having difficulty in controlling their trunk positions. The BWS system consists of an actuator module, a winch, and a transfer module as shown in Fig. 1.

Since the weight of the exoskeleton imposes physical burden to the subjects wearing the exoskeleton robot and adverse effect on the gait [27], a gravity compensator is designed to support the weight of the exoskeleton. The weight of the exoskeleton is supported by linear springs instead of counter masses to reduce the inertial effect on the exoskeleton. Using a 4-bar linkages and cable, the supporting force generated by the springs remains constant regardless of the position of the exoskeleton.

## Method

This study was a randomized [1:1], single-blind, parallel-group study conducted at Asan Medical Center (AMC) in Seoul, South Korea from December 2018 to June 2019. The study was approved by the Asan Medical Center Institutional Review Board (IRB, No. 2018-1254).

### Participants

The minimum number of subjects needed for statistical analysis was determined to be 10 for each group. Considering the drop-out rate, 24 stroke patients were recruited. All participants were informed of the study objectives and procedures prior to signing an informed consent form. The inclusion criteria were: (1) Adult who is over 19 years old; (2) Being able to board the Healbot T; (3) Functional Ambulation Category (FAC) is higher than or equal to 2 (0 = Patient cannot walk, 5 = Patient can walk independently anywhere); (4) Being able to walk independently before onset. The exclusion criteria were: (1) Difficulties in communication due to serious cognitive or speech disorders; (2) Limited range of motion; (3) Ulcers that have bedsores, skin diseases, wounds or unhealed ulcers at the area touching cuff or harness; (4) Uncontrolled hypertension or orthostatic hypotension; (5) Inability to tolerate robot walking due to cardiovascular disease, heart failure, malignant disease, lung disease, nervous system disease, diabetes, etc; (6) Severe osteoporosis; (7) Severe deformity or pain in the legs; (8) Severe mental illness, neuropathy or being significantly less cooperative; (9) Pregnancy; (10) Severe stiffness over Modified Ashworth Scale 3 (0 = No increase in muscle tone, 4 = Affected side(s) rigid flexion or extension); (11) Tremor; (12) Weight is more than 100 kg; (13) Height is less than 150 cm or more than 185 cm; (14) Participated in a rehabilitation robot study for stroke within 30 days; (15) In addition, if the researchers determine that participation in this study is inappropriate. (e.g., the participant’s disobeying to the instructions given by the researchers.)

Detailed information on the subjects is listed in Table 1. Independent T-test (2-side) revealed that there were no statistical differences in weight, age, duration of onset, and FAC between both groups. However, the height of the pelvis-on group is greater than the pelvis-off group ($\hbox {p}<0.05$).

**Table 1:** Participant information

	Age	Gender	Weight	$\hbox {Height}^{*}$	Affected	Stroke	Onset	FAC	Note
	(y)		(kg)	(cm)	side	etiology	(y)		
Pelvis-off	64	F	59	150	L	Infarction	13	5	
(N = 12)	84	M	66	167	L	Hemorrhage	2	2	DO
	57	F	73	170	R	Hemorrhage	15	4	
	55	F	77	155	L	Infarction	16	4	
	74	M	62	160	L	Infarction	7	3	
	72	M	66	165	R	Hemorrhage	14	4	
	65	F	60	160	R	Infarction	11	4	
	56	M	80	167	L	Infarction	17	3	
	65	F	58	151	L	Infarction	11	5	
	65	F	52	150	R	Hemorrhage	18	4	
	67	M	68	153	L	Infarction	24	4	
	62	F	58	157	R	Hemorrhage	8	4	
Pelvis-on	55	M	77	180	R	Infarction	7	6	
(N = 12)	66	M	73	165	L	Infarction	19	4	
	65	M	59	165	L	Infarction	6	6	
	62	M	65	164	L	Hemorrhage	6	6	
	57	M	72	165	R	Infarction	13	6	
	63	M	62	166	R	Infarction	30	4	
	71	M	71	164	L	Infarction	23	4	
	53	M	65	164	R	Hemorrhage	9	4	
	65	M	66	167	R	Infarction	14	5	
	75	M	79	172	R	Infarction	6	4	
	81	F	52	150	L	Infarction	24	3	
	62	M	77	166	R	Hemorrhage	13	3	

DO stands for drop-out. $^{*}$ indicates significant difference between two groups with $p<0.05$

### Protocol

**Fig. 3:**
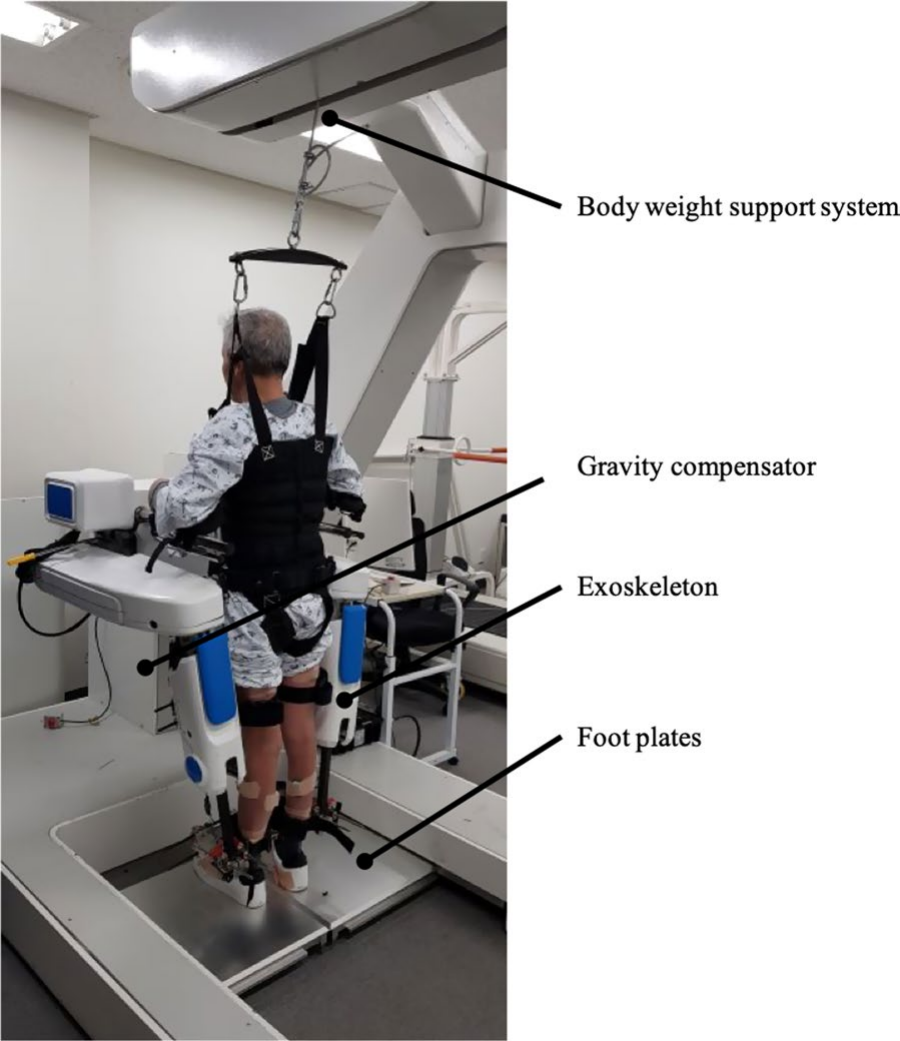
Subject in the Healbot T during training

Subjects with hemiparesis from stroke visited AMC 10 times within one month. During a visit, subjects were asked to walk in Healbot T. Figure 3 shows a subject in the Healbot T during a gait training.

The subjects were randomly assigned into two groups, which were pelvis-off and pelvis-on groups. For allocation of the subjects, a random sequence was generated by a computer using SAS (Ver. 9.4, SAS Institute). The random sequence type was set as blocked randomization with groups of the same size. For the pelvis-off group, pelvic movements in the transverse plane were not allowed during gait trainings, whereas translational and rotational pelvic movements in the transverse plane were provided for the pelvis-on group. The data analyst was kept blind to the interventions of the subjects. Although the subjects were not informed to which group they were assigned, some of the subjects might have noticed the differences in pelvic movements provided by Healbot T during training.

Prior to the gait trainings, the length of the body segments of each subject was measured and used to adjust the length of the corresponding link of the exoskeleton. Each training session lasted 30 minutes, during which the subjects walked in Healbot T. The exoskeleton was attached to the subjects using braces and straps at the pelvis, thighs, and calves. If necessary, the BWS system was used to support the weight of the subjects. Predefined trajectories, which were collected from a healthy person on a treadmill prior to this experiment, were used to control the exoskeleton. Note that the trajectories of the pelvic joints for the pelvis-off group were set to be constant. At the beginning of each session, preferred walking speeds were selected by the subjects. In order to select the preferred walking speed, while walking in Healbot T at the initial walking speed of 0.5 km/h, the subjects verbally expressed their wish to increase or decrease the walking speed, to which the operator set the walking speed of Healbot T, until they found a comfortable speed.

**Fig. 4:**
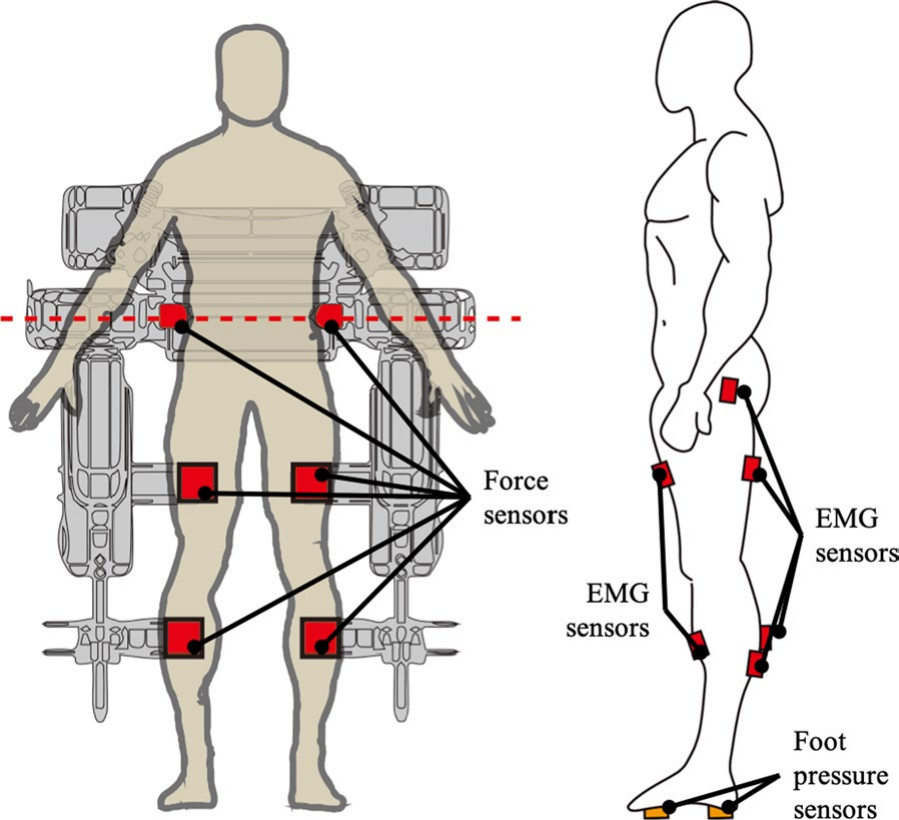
Locations of sensors. 6 force sensors are at the pelvis, thigh, and calf of each side. 12 EMG sensors are at the gluteus medius, rectus femoris, biceps femoris, tibialis anterior, gastrocnemius medial, gastrocnemius lateral. 2 pressure sensors are beneath each foot

Interaction forces between the subjects and the exoskeleton were measured using force sensors (UMM31-K100, Dacell) installed at the braces during the trainings. Muscle activation of the gluteus medius (Gmed), rectus femoris (RF), tibialis anterior (TA), biceps femoris (BF), gastrocnemius medial (GCM-M), gastrocnemius lateral (GCM-L) muscles of both sides were measured using EMG sensors (Trigino, Delsys) during the trainings in Healbot T on the 1st, 5th, and 10th day. Two custom-made foot pressure sensors using force sensitive resisters (FSR-402, Interlink) were attached beneath each sole to determine the gait phase. See Fig. 4 for the locations of the sensors. Gait parameters such as stride length, cadence, and walking speed were measured using OptoGait (Microgate) on the 1st, 5th, and 10th day after the trainings followed by a 20-min cool-down period.

## Result

The subjects were recruited from 7 December 2018 to 18 June 2019. Eligible participants were randomly assigned to the pelvis-off ($\hbox {n}=12$) and pelvis-on ($\hbox {n}=12$) groups. The subjects visited AMC 10 times within one-month after random assignment. After the 4th day, however, one subject in the pelvis-off group had dropped out because of personal reasons. No unintended effects were reported during the experiment.

### Statistical analysis

Due to one drop-out from the pelvis-off group, data from 11 subjects were available whereas data from 12 subjects of the pelvis-on group were used for analysis. Normality of the measured data, which were the interaction forces and EMG signals data averaged over steps, self-selected walking speed, and gait parameters, were checked using Shapiro-Wilk test. One-way Repeated Measured (RM) ANOVA was used for longitudinal study on data collected on 1st, 5th and 10th day with significance level set to be $p < 0.05$ and denoted by * mark. For multiple comparison, Bonferroni correction was used and significant differences are denoted by a (1st vs 5th day), b (5th vs 10th day), and c (1st vs 10th day) with $p<0.01667$. Independent T-test (2-side) was used for comparison of both groups. Significance level of independent T-test (2-side) was set to be $p < 0.05$ and denoted by $^+$ mark.

### Self-selected speed

**Fig. 5:**
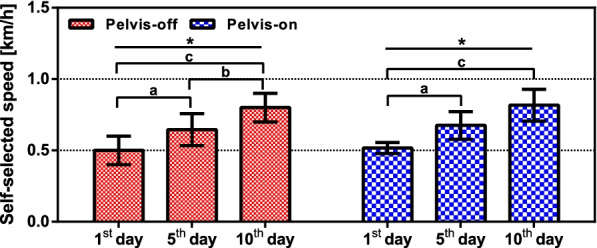
The self-selected speed of both groups. The bar graphs represent mean of self-selected speed in both groups. The error bars represent standard deviation of self-selected speed in both groups. One-way RM ANOVA result: *($\hbox {p}<0.05$). Bonferroni correction: **a** (1st and 5th), **b** (5th and 10th), **c** (1st and 10th)

The measured self-selected walking speeds on 1st, 5th, and 10th day are shown in Fig. 5. The walking speeds have no significant differences between two groups. However, each group exhibited increased self-selected walking speed as training progressed. A pairwise comparison with Bonferroni adjustment revealed a significant increase in all data pairs (1st and 5th day with $\hbox {p} = 0.00023$, 5th and 10th day with $\hbox {p} = 0.00015$, 1st and 10th day with $\hbox {p} < 0.00001$) of the pelvis-off group and the data pair of 1st and 5th day ($\hbox {p} = 0.0008$) and 1st and 10th day ($\hbox {p} = 0.00005$) of the pelvis-on group.

### Interaction force

The interaction forces were measured using the force sensors installed on the braces at pelvis, thighs, and calves as shown in Fig. 4. Note that two force sensors were required to measure the interaction force at the pelvis since unilateral force (i.e. pushing against the sensor) was applied to the sensors. Therefore, the net interaction forces were calculated as $f_{\mathrm{{pelvis}}} = f_{\mathrm{{A}}} + f_{\mathrm{{U}}}$, where $f_{\mathrm{{A}}}$ represented the interaction force at the affected side (A) of the pelvis and $f_{\mathrm{{U}}}$ denoted the interaction force at the unaffected side (U).

**Fig. 6:**
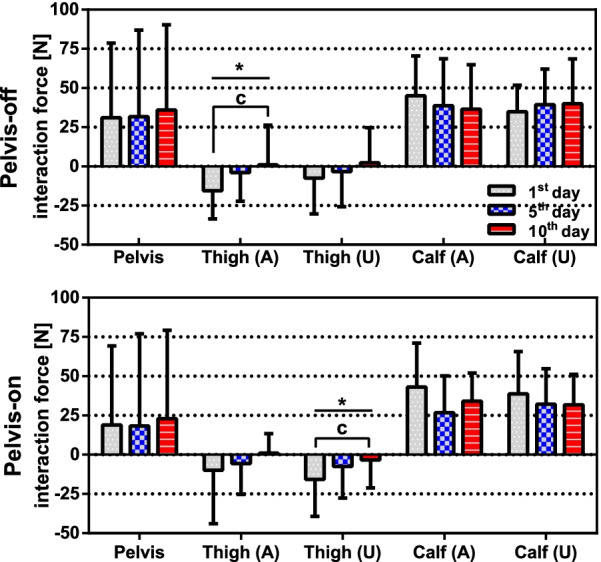
Measured interaction forces. The bar graphs represent mean of interaction force in both groups. The error bars represent standard deviation of interaction forces in both groups. One-way RM ANOVA result: *($\hbox {p}<0.05$). Bonferroni correction: **a** (1st and 5th), **b** (5th and 10th), **c** (1st and 10th)

Mean of interaction forces were obtained by averaging interaction forces of each subject, which were averaged over gait cycles. The interaction forces are shown in Fig. 6. No significant change in interaction forces of the both groups was found except for the thigh in the affected side of the pelvis-off group and the thigh in the unaffected side of the pelvis-off group. When Bonferroni correction was used for multi-comparison, the interaction force at the thigh in the affected side of the pelvis-off group decreased by 93.3% from 1st to 10th day (p = 0.01432) and that of the thigh in the unaffected side of the pelvis-on group decreased by 79.0% from 1st to 10th day (p = 0.00698). Positive interaction forces at the pelvis indicated that the patients leaned toward the affected side, which were observed in both groups.

### EMG signal

The measured EMG signals were filtered with a band pass filter with pass band frequencies of $20\sim 500$ Hz, rectified, and normalized by their maximum values. Mean of EMG was calculated by averaging the EMG signals of each subject, which were obtained by averaging measured EMG signals over gait cycles.

**Fig. 7:**
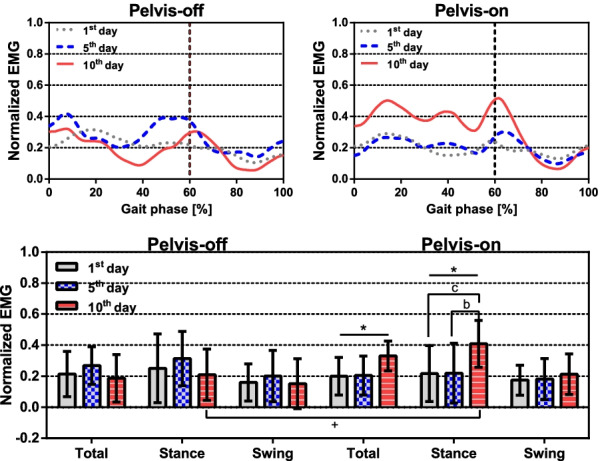
Normalized EMG records of Gmed on the affected side of both groups are at the top. Vertical dashed lines indicate the toe-off. The EMG records during different gait phases are at the bottom. The error bars represent standard deviation of EMG signal. One-way RM ANOVA result: *($\hbox {p}<0.05$). Bonferroni correction: **a** (1st and 5th), **b** (5th and 10th) and **c** (1st and 10th). Independent T-test (2-side) result: $^{+}$ ($\hbox {p}<0.05$)

As shown in Fig. 7, there is no significant difference in EMG signals between both of the groups on the 1st day. However, the mean EMG signals of the pelvis-on group increased by 46.8% on the last day of training. Multiple comparison with Bonferroni correction showed 88.5% increase in the Gmed EMG signal during the stance phase from 1st to 10th day (p = 0.00384) and 86.5% increase between 5th and 10th day (p = 0.00373) in the pelvis-on group. EMG signal of gluteus medius during the stance phase in the pelvis-on group were greater than the pelvis-off group at 10th day.

**Fig. 8:**
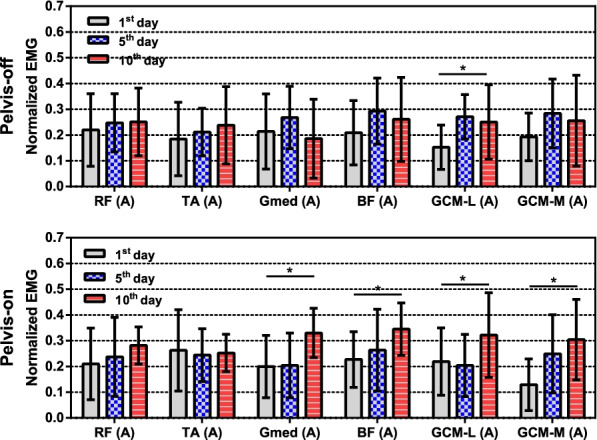
Averaged EMG signals of the 6 muscles on the affected sides of both groups. Top is the EMG signals of the pelvis-off group and bottom is the EMG signal of the pelvis-on group. One-way RM ANOVA result: *($\hbox {p}<0.05$)

The mean EMG signals of 3 other muscles in the affected side of the pelvis-on group, which were BF, GCM-L and GCM-M, increased by 51.9% ($\hbox {p}<0.05$), 65.3% ($\hbox {p}<0.05$) and 135.7% ($\hbox {p}<0.05$) during the experiment as shown in Fig. 8. In the pelvis-off group, the EMG signal of GCM-L increased by 64.4% ($\hbox {p}<0.05$) on the 10th day.

### Gait parameters

**Fig. 9:**
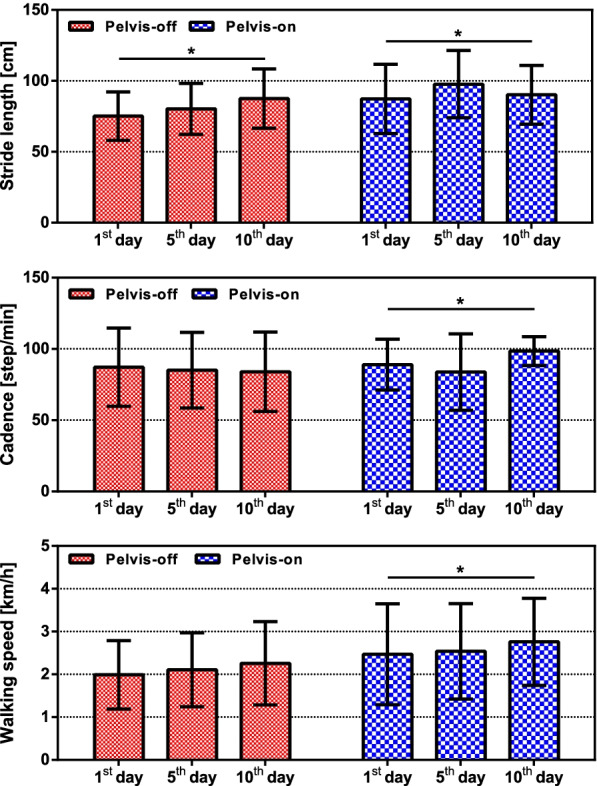
Measured gait parameters. Red and blue bars represent the pelvis-off group and the pelvis-on group. Error bars showed standard deviation. One-way RM ANOVA result: *($\hbox {p}<0.05$)

Figure 9 shows the measured gait parameters which were stride length, cadence and walking speed. In order to measure the gait parameters, the patient independently walked on the ground without Healbot T while measuring the gait parameters using OptoGait. Stride length of both pelvis-off and pelvis-on group increased by 16.5% and 3.3%, respectively. Furthermore, cadence and walking speed of the pelvis-on group showed 10.6% and 11.8% increases.

## Discussion

The effect of pelvic movements during gait training was investigated. In order to provide gait patterns including pelvic movements, a robotic gait training system, which was called Healbot T and capable of pelvic movements, was developed. 24 subjects, one of whom had dropped out due to personal reasons, participated in the experiment and were randomly assigned into two groups. The pelvis-on group was provided with pelvic movements whereas no pelvic movement was given to the pelvis-off group during gait trainings in Healbot T.

It was hypothesized that since one of the reasons for the interaction forces was the limited degrees of freedom of the exoskeleton, constrained pelvic movements imposed by the exoskeleton resulted in higher interaction forces at the pelvis. Therefore, the rotational and lateral pelvic movements contributed to reduce the interaction forces as reported in [28]. However, no significant difference of interaction forces was measured between the pelvis-on and pelvis-off group. Therefore, it was not clearly shown that lateral and rotation pelvic movement contribute to reduction of interaction forces. Positive values of the interaction forces indicated that both group leaned toward affected sides, which might be the result of poor trunk control during the trainings.

Muscle activation of gluteus medius (Gmed) in Fig. 7 showed no significant difference between 1st and 10th days in the pelvis-off group whereas the pelvis-on group showed increased muscle activation, which was not consistent with what was reported in the previous study [24]. The difference might be caused by the trajectories with larger lateral displacement provided by the exoskeleton. In the pelvis-off group, Gmed muscles were activated around toe-off, which was not observed in the experiments with healthy subjects [21, 22, 24, 29] but consistent with the experiments with stroke patients by van Kamman [30]. The muscle activation around toe-off of the swing leg might occur due to the effort to create the circumduction, which was often observed in the gait patterns of the stroke patients or weight shifting during the double stance phase since Gmed contributes to weight shifting [31].

No significant difference in Gmed muscle activation in the pelvis-off group was observed whereas increased muscle activation in the pelvis-on group were found during the stance phase, which is consistent with what was reported [29, 30, 32]. This implies that the muscle activation occurred due to voluntary balancing and weight bearing during the stance phase. Therefore, it is important to have lateral pelvic movements in gait training to improve balancing and weight shifting since Gmed muscles take a critical role in balancing and weight shifting during walking [33].

In the previous research by Hidler and Wall [29], increased activation in quadriceps muscles such as rectus femoris and gluteus muscle groups and decreased activation in gastrocnemius and tibialis anterior muscles were reported. However, in this study, RF and TA muscles of both groups showed no significant changes whereas other muscles except RF and TA showed increased activation in the pelvis-on group as shown in Fig. 8. Since increased EMG signals indicates more voluntary muscle activation [34], increased muscle activation of the pelvis-on group implies voluntary muscle activation to support body weight with the affected leg during the trainings with pelvic motions.

Increased stride length of both groups and cadence of the pelvis-on group are partially consistent with Veneman [35] who reported increased step length and decreased cadence of walking with fixed pelvis. The discrepancies might come from the fact that the walking speed was fixed in [35] whereas the walking speed in this study was self-selected.

The findings in this study suffer from small number of the subjects, although the experimental results showed positive effects of the pelvic movements during gait training. Since this study was designed as preliminary clinical test, the experiment was carried out with small number of the subjects. Confirmatory clinical trial is planned in the future with a larger number of subjects.

Although the gait parameters measured 20-minute after gait training on 1st, 5th, and 10th day exhibited increased stride length in both groups as well as increased cadence and walking speed in the pelvis-on group, it is not clear if the gait training has long-term effects on the functionality of the participants. Furthermore, the trajectories used in the training was obtained from a person without hemiparesis. It was not clear if it was optimal to use the unimpaired gait patterns in the training for maximum retention of the effect of the trainings.

## Conclusion

A robotic gait training system capable of providing pelvic movement, which was called Healbot T, was developed. The effects of translational and rotational pelvic movements on gait training of stroke patients were studied using Healbot T. 23 out of 24 subjects successfully finished the experiment. The subjects were randomly assigned into pelvis-off and pelvis-on groups. The pelvis-off group was trained without pelvic movements while the pelvis-on group was provided with the pelvic movements during gait training using Healbot T. No subject experiencing any adverse event was reported in this study. Although no significant difference of interaction force of pelvis was observed in the both group, muscle activation of Gmed as well as BF, GCM-M and GCM-L muscles increased in the pelvis-on group. These results indicated that the pelvic motions provided by Healbot T induced voluntary muscle activation of the subjects for weight bearing. Recovering of weight bearing ability in the pelvis-on group is considered to improve gait parameter such as cadence and walking speed. Assistance of pelvic movement by Healbot T shows potential to recovery patient’s walking.

## Data Availability

The datasets used and analyzed in the manuscript are available from the corresponding author on reasonable request (Email: junhochoi@kist.re.kr).
